# Immune Responses to *Mycobacterium tuberculosis* Infection in the Liver of Diabetic Mice

**DOI:** 10.3390/biomedicines12061370

**Published:** 2024-06-20

**Authors:** Ali Badaoui, Kayvan Sasaninia, Aishvaryaa Shree Mohan, Abrianna Beever, Nala Kachour, Anmol Raien, Afsal Kolloli, Ranjeet Kumar, Santhamani Ramasamy, Selvakumar Subbian, Vishwanath Venketaraman

**Affiliations:** 1 College of Osteopathic Medicine of the Pacific, Western University of Health Sciences, Pomona, CA 91766, USA; ali.badaoui@westernu.edu (A.B.); kayvan.sasaninia@westernu.edu (K.S.); amohan@westernu.edu (A.S.M.); anmol.raien@westernu.edu (A.R.); 2 College of Osteopathic Medicine, Kansas City University, Kansas City, MO 64106, USA; 3 College of Natural and Agricultural Science, University of California Riverside, Riverside, CA 92521, USA; 4 Public Health Research Institute, New Jersey Medical School, Rutgers University, Newark, NJ 07103, USA; ak1482@njms.rutgers.edu (A.K.); rk879@njms.rutgers.edu (R.K.); santhuvet@gmail.com (S.R.); subbiase@njms.rutgers.edu (S.S.)

**Keywords:** tuberculosis, diabetes, hepatic tuberculosis, host immune response, cytokine imbalance, redox imbalance, tuberculosis pathology, disease score, granuloma

## Abstract

Individuals with uncontrolled diabetes are highly susceptible to tuberculosis (TB) caused by *Mycobacterium tuberculosis* (*M. tb*) infection. Novel treatments for TB are needed to address the increased antibiotic resistance and hepatoxicity. Previous studies showed that the administration of liposomal glutathione (L-GSH) can mitigate oxidative stress, bolster a granulomatous response, and diminish the *M. tb* burden in the lungs of *M. tb*-infected mice. Nonetheless, the impact of combining L-GSH with conventional TB treatment (RIF) on the cytokine levels and granuloma formation in the livers of diabetic mice remains unexplored. In this study, we evaluated hepatic cytokine profiles, GSH, and tissue pathologies in untreated and L-GSH, RIF, and L-GSH+RIF treated diabetic (db/db) *M. tb*-infected mice. Our results indicate that treatment of *M. tb*-infected db/db mice with L-GSH+RIF caused modulation in the levels of pro-inflammatory cytokines and GSH in the liver and mitigation in the granuloma size in hepatic tissue. Supplementation with L-GSH+RIF led to a decrease in the *M. tb* burden by mitigating oxidative stress, promoting the production of pro-inflammatory cytokines, and restoring the cytokine balance. These findings highlight the potential of L-GSH+RIF combination therapy for addressing active EPTB, offering valuable insights into innovative treatments for *M. tb* infections.

## 1. Introduction

Tuberculosis (TB), caused by the bacterial infectious agent *Mycobacterium tuberculosis* (*M. tb*), continues to be a significant global health challenge [1]. *M. tb*, believed to be over 3 million years old, has a long history, dating back to ancient Greece and Rome [2]. In the early 20th century, TB, formerly referred to as consumption, was the leading cause of death in the US [3]. Untreated TB can be fatal, with over 1 million deaths reported worldwide in 2022 alone [1]. It ranks as the second-most deadly infectious disease, following COVID-19, and is the 13th rising cause of death globally [1]. 

*M. tb* is responsible for more than 10 million active TB cases annually worldwide [1]. Individuals with uncontrolled diabetes mellitus and HIV/AIDS face a higher risk of contracting tuberculosis due to their compromised immune systems [1]. Moreover, diabetic individuals with untreated latent *M. tb* infection are more prone to developing TB disease compared to those without diabetes [1]. 

The transmission of *M. tb* primarily occurs through the inhalation of aerosol droplets containing *M. tb* into the lungs, making pulmonary TB the most abundant form of the disease [4]. However, *M. tb* can also be disseminated to other organs, such as the liver [4].

Hepatic TB is estimated to occur in approximately 1% of active tuberculosis cases, with 79% attributed to micronodular-miliary tuberculosis and 21% to macronodular-local hepatic tuberculosis. The bacilli enter the liver through the hepatic artery in miliary (micronodular) tuberculosis and the portal vein in macronodular tuberculosis [5]. 

Prior studies have demonstrated that glutathione (GSH) plays a significant role in controlling *M. tb* infection by exerting direct antimycobacterial effects and enhancing natural killer and T-cell responses against *M. tb* infection [6]. The depletion of GSH results in immune dysregulation and exacerbates *M. tb* infection, particularly in immunocompromised individuals. The treatment of *M. tb*-infected diabetic (db/db) mice with a combination of rifampicin (RIF) and liposomal GSH (L-GSH) resulted in the maximum reduction in *M. tb* burden in the lungs four weeks post-infection and treatment [7]. 

In this study, we aimed to characterize the effects of L-GSH+RIF treatment on liver immunopathology in diabetic mice infected with *M. tb*. We evaluated the cytokine levels and histopathological changes in the liver of *M. tb*-infected db/db mice treated with sham, L-GSH, RIF, and RIF+L-GSH.

## 2. Materials and Methods

### 2.1. Bacteria and Chemicals 

As previously mentioned, the *M. tb* H37Rv strain was cultivated in vitro and subjected to infection procedures [8]. The bacteria grew to OD600 = 0.6 to 0.8 in Middlebrook 7H9 medium (Difco BD, Franklin Lakes, NJ, USA) with 10% albumin dextrose complex (ADC) enrichment and were then kept frozen at −80 °C in aliquoted stocks. As described before [9], the thawed stock vials were diluted to create the inoculum for infection. Unless otherwise noted, all compounds were acquired from Millipore Sigma (Millipore Sigma, Burlington, MA, USA).

### 2.2. Aerosol Infection of Mice, Treatment, and Bacterial CFU Assay 

Six- to eight-week-old diabetic C57BL db/db mice were bought from Jackson Laboratories (Bar Harbor, ME, USA) and infected with the *M. tb* inoculum according to the prior instructions [10]. Using a Madison Chamber (Glas-Col LLC, Madison, WI, USA) designed to administer a standard low dose of roughly 100 CFU, mice were exposed to *M. tb* aerosols as previously reported [7,11]. Three hours after infection (T = 0), three mice were sacrificed to estimate the number of bacteria implanted in the liver. After extraction, the livers were homogenized in 2 mL of sterile 1XPBS, serially diluted, and plated on Middlebrook 7H11 agar media (Difco BD, Franklin Lakes, NJ, USA) [12]. About 40% of the liver was used in this process. 

Following infection, mice were randomized into four groups: (1) no therapy, (2) 40 mM L-GSH treatment, (3) RIF treatment, and (4) combination treatment (RIF plus 40 mM L-GSH treatment). Treatments were administered beginning on the day of infection and lasting until the trial endpoint, which was eight weeks after infection. L-GSH was given through drinking water, which was replaced every three days. From day 4 to 8 weeks post-infection, RIF was given orally three times a week at a dose of 8 mg/kg. Three mice from each group were euthanized at predetermined intervals (4 and 8 weeks post-infection), and along with a standard autopsy, the liver was removed per the previous instructions [12,13,14,15,16]. 

Previous studies involving mouse model TB experiments have consistently terminated at the 4-week and 8-week time points, as they provide a comprehensive and comparable view of the disease progression [13,14,15,16]. The 4-week time point provides insight into the acute phase of bacterial burden, while the 8-week time point demonstrates the chronic phase [13]. Additionally, terminating at 4 and 8 weeks allows for assessing both the early and long-term effects of treatments and ensures ethical treatment of the animals.

The lower lobe of the liver was preserved in 10% neutral-buffered formalin for histological investigations. After passing through a 0.2-micron filter, lung lysates were utilized for further GSH and cytokine assays.

### 2.3. Histology Staining of Liver Sections and Morphometry

To visualize the organization of granulomas and distribution of leukocytes, portions of the liver fixed in 10% neutral formalin solution were paraffin-embedded, cut into 5 µm slices, and stained with Hematoxylin-Eosin (H&E). An Olympus Model BX41TF microscope was utilized for analysis of the stained sections and an Olympus DP Controller for capturing photographs. 

The H&E-stained mouse liver sections were analyzed using Path Scan Enabler-5 (Mayer Scientific, Houston, TX, USA) for morphometric analysis of granuloma and immune cell infiltration. This was followed by morphometric determination of the liver area involved in the granulomatous response using Sigma Scan Pro (Systat Software Inc., San Francisco, CA, USA, https://www.lumafield.com/), as previously mentioned [17]. The Scheuer system was used to assess the disease score of the liver sections, graded as follows: Grade 1: minimal inflammation without necrosis, Grade 2: inflammation with mild and focal necrosis, Grade 3: moderate necrosis and severe inflammation, and Grade 4: severe necrosis and severe inflammation [18]. Disease scores were assigned for the treated and untreated livers of db/db mice infected with *M. tb*.

### 2.4. Quantification of Glutathione Levels 

The Glutathione Colorimetric Detection Kit from Invitrogen, Carlsbad, CA, USA (Cat. EIAGSHC) was utilized to measure the GSH levels following the manufacturer’s protocol (Thermo Fisher Scientific, Waltham, MA, USA). The GSH levels were determined for the liver tissue homogenates of diabetic mice treated with 40 mM L-GSH, RIF, and a combination of RIF and 40 mM L-GSH at 4 weeks and 8 weeks post-infection. The oxidized GSH (GSSG) was subtracted from the total GSH to provide the reduced GSH (rGSH). The data were expressed in μM GSH per μg of protein, and all measurements were normalized to the total protein levels in the samples.

### 2.5. Cytokine Measurement 

Using enzyme-linked immunosorbent assay (ELISA) kits, the levels of IL-12, IFN-γ, IL-2, TNF-α, IL-17, IL-6, and TGF-β1 were assessed in the liver tissue homogenates from untreated, 40 mM L-GSH-, RIF-, and a combination of RIF and 40 mM L-GSH-treated mice. These kits included the IL-12 p70 Mouse Uncoated ELISA Kit (Cat. 88–7,121–88), IFN-γ Mouse Uncoated ELISA Kit (Cat. 88–7,314–88), IL-2 Mouse Uncoated ELISA Kit (Cat. 88–7,024–88), TNF-α Mouse Uncoated ELISA Kit (Cat. 88–7,324–88), IL-17A (homodimer) Mouse Uncoated ELISA Kit (Cat. 88–7,371–86), IL-6 Mouse Uncoated ELISA Kit (Cat. 88–7,064–88), and Human/Mouse TGF-β1 Uncoated ELISA Kit (Cat. 88–8,350–88). Thermo Fisher Scientific provided all the ELISA kits, and the manufacturer’s instructions were followed when measuring all the cytokine levels. The data were expressed in pg of cytokine per μg of protein, and all values were normalized to the total protein levels in the samples. 

### 2.6. Statistical Analysis 

For statistical analysis, GraphPad Prism Software (Ver. 8) was used, and when comparing multiple groups, a one-way ANOVA with Brown–Forsythe and Welch correction was applied. *p*-values less than 0.05 were regarded as statistically significant. All presented numbers are the means and standard deviations for each category. An asterisk (*) indicates that the item was directly compared to the preceding category. When two asterisks are shown (**), a *p*-value of less than 0.01 is presumed. When three asterisks (***) are used, it is assumed that the *p*-value is less than 0.005, and when four asterisks are shown (****), a *p*-value of less than 0.0001 is presumed.

## 3. Results

### 3.1. Levels of Cytokines and GSH in Liver Lysates at 4 Weeks Post-M. tb Infection and Antibiotics with or without GSH Treatment

**Figure 1:**
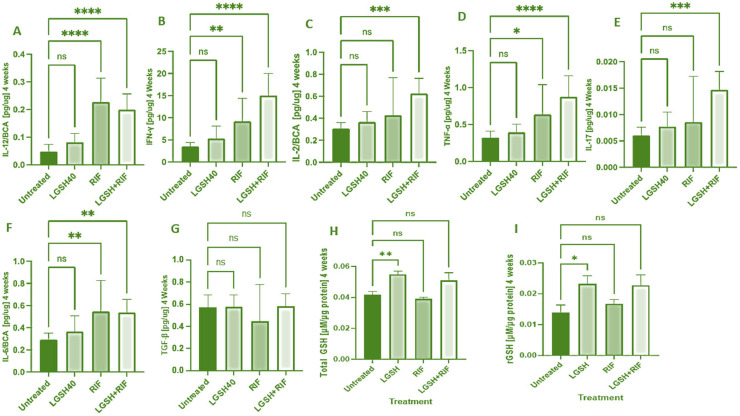
Levels of IL-12 (**A**), IFN-γ (**B**), IL-2 (**C**), TNF-α (**D**), IL-17 (**E**), IL-6 (**F**), TGF-β (**G**), total GSH (**H**), and rGSH (**I**) in liver lysates of *M. tb*-infected mice at 4 weeks post-infection. We used GraphPad Prism Software 8 for statistical analysis. The graphs show the values of the mean ± standard deviation for each group, with n = 6 per group per time point. One-way ANOVA was utilized in analyzing the data for multiple groups. *p*-values of <0.05 (*), <0.01 (**), <0.005 (***), and <0.0001 (****) were considered significant. *p*-values of >0.05 were considered not significant (ns). The results shown are an average of six mice.

At 4 weeks, most treatments showed an increase in the IL-12, IFN-γ, IL-2, TNF-α, IL-17, IL-6, TGF-β, and GSH levels when compared to the untreated group (Figure 1). LGSH40 treatment showed a non-significant increase in the IL-12, IFN-γ, IL-2, TNF-α, IL-17, IL-6, and TGF-β levels when compared to untreated mice at 4 weeks post-infection (Figure 1A–G). LGSH40 treatment resulted in a significant increase in the total and reduced GSH, (Figure 1H, I), while RIF treatment resulted in a significant increase in IL-12, IFN-γ, TNF-α, and IL-6 production when compared to untreated mice at 4 weeks post-infection (Figure 1A,B,D,F). LGSH+RIF resulted in a significant increase in IL-12, IFN-γ, IL-2, TNF-α, IL-17, and IL-6 production when compared to untreated mice at 4 weeks post-infection (Figure 1A–F). Higher levels of IFN-γ, IL-2, TNF-a, IL-17, and the total and reduced forms of GSH were observed in the L-GSH+RIF treatment group when compared to the lone RIF treatment group (Figure 1B–D,H,I).

### 3.2. Levels of Cytokines and GSH in the Liver Lysates at 8 Weeks Post-Infection and Treatment

**Figure 2:**
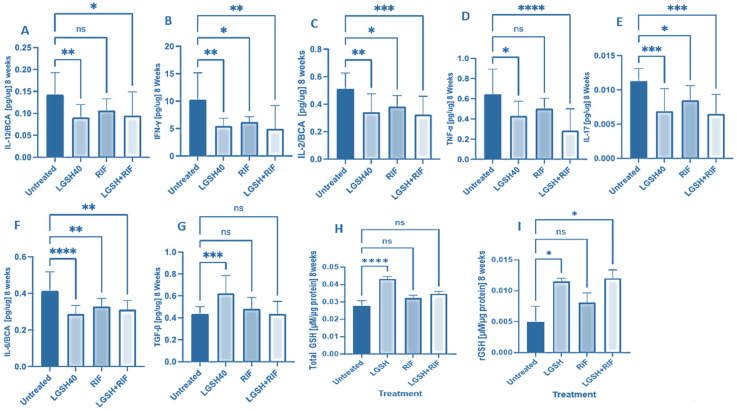
Levels of IL-12 (**A**), IFN-γ (**B**), IL-2 (**C**), TNF-α (**D**), IL-17 (**E**), IL-6 (**F**), TGF-β (**G**), total GSH (**H**), and rGSH (**I**) in liver lysates of *M. tb*-infected mice at 8 weeks post-infection. We used GraphPad Prism Software 8 for statistical analysis. The graphs show the values of the mean ± standard deviation for each group, with n = 6 per group per time point. One-way ANOVA was utilized in analyzing the data for multiple groups. *p*-values of <0.05 (*), <0.01 (**), <0.005 (***), and <0.0001 (****) were considered significant. *p*-values of >0.05 were considered not significant (ns). The results shown are an average of six mice.

At 8 weeks, all treatments showed a decrease in IL-12, IFN-γ, IL-2, TNF-α, IL-17, IL-6, TGF-β, and GSH production (Figure 2). LGSH40 treatment resulted in a significant decrease in IL-12, IFN-γ, IL-2, TNF-α, IL-17, and IL-6 production compared to untreated mice at 8 weeks post-infection (Figure 2A–F). LGSH40 treatment resulted in a significant increase in TGF-β and the total and reduced GSH production compared to untreated mice at 8 weeks post-infection (Figure 2G–I). RIF treatment resulted in a significant decrease in IFN-γ, IL-2, IL-17, and IL-6 production compared to untreated mice at 8 weeks post-infection (Figure 2B,C,E,F). LGSH+RIF resulted in a significant decrease in IL-12, IFN-γ, IL-2, TNF-α, IL-17, and IL-6 production compared to untreated mice at 8 weeks post-infection (Figure 2A–F). LGSH+RIF resulted in a significant increase in reduced GSH (rGSH) compared to untreated mice at 8 weeks post-infection (Figure 2I). When compared to the lone RIF group, mice treated with L-GSH+RIF had a higher increase in the levels of reduced forms of GSH (Figure 2I) and a further diminishment in the levels of IL-12, IFN-γ, IL-2, TNF-α, IL-17, and IL-6 (Figure 2A–F).

### 3.3. Effect of GSH with or without RIF on the Liver Pathology of M. tb-Infected db/db Mice

To determine the effectiveness of GSH with or without RIF on TB pathology in the liver, we performed a histologic analysis of treated and untreated *M. tb*-infected db/db mice.

**Figure 3:**
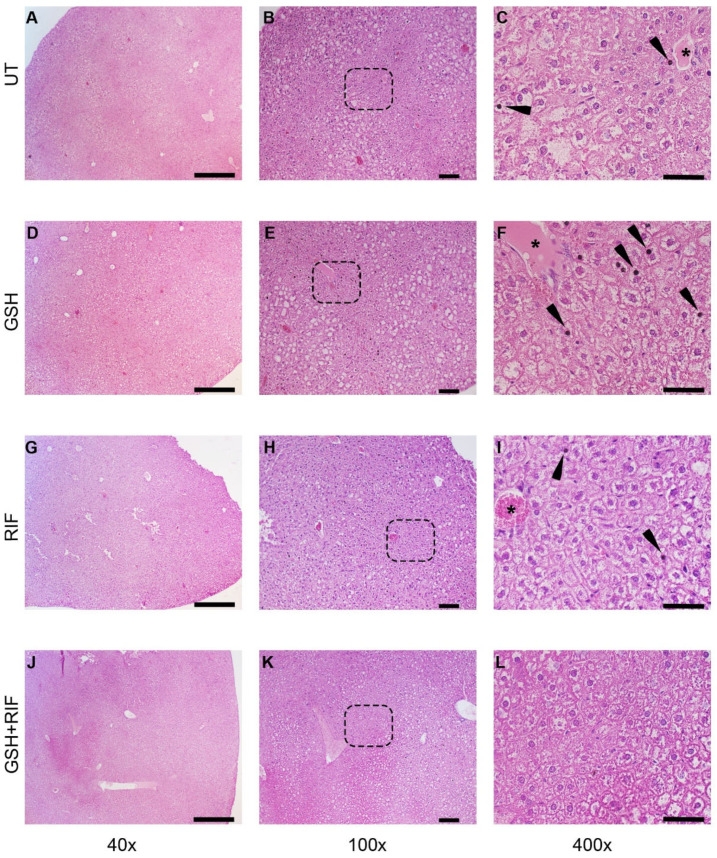
Histopathology of the livers of untreated and treated *M. tb*-infected db/db mice at 4 weeks post-infection/treatment. (**A**–**C**) Untreated (UT); (**D**–**F**) glutathione-treated (GSH); (**G**–**I**) rifampicin-treated (RIF); (**J**–**L**) glutathione plus rifampicin-treated (GSH+RIF). Images (**A**,**D**,**G**,**J**) are at 40× magnification; (**B**,**E**,**H**,**K**) are 100× magnification; (**C**,**F**,**I**,**L**) are 400× magnification. Arrowheads indicate pyknotic nuclei, and * refers to the hepatic veins with various levels of disorganization. The dotted rectangles in (**B**,**E**,**H**,**K**) refer to the enlarged sections shown in (**C**,**F**,**I**,**L**), respectively. The scale bar in (**A**,**D**,**G**,**J**) is 500 µm; (**B**,**E**,**H**,**K**) is 100 µm; (**C**,**F**,**I**,**L**) is 50 µm.

At 4 weeks post-infection/treatment, broadly inflammatory cellular infiltration was noted in *M. tb*-infected db/db mice livers, regardless of the treatment (Figure 3). Liver sections from all animal groups had hepatocytes with vacuolated cytoplasm. Animals without any treatment or those treated with GSH or RIF had pyknotic nuclei scattered in the liver parenchyma; a very scanty distribution of these structures was noted in the GSH+RIF treatment group.

**Figure 4:**
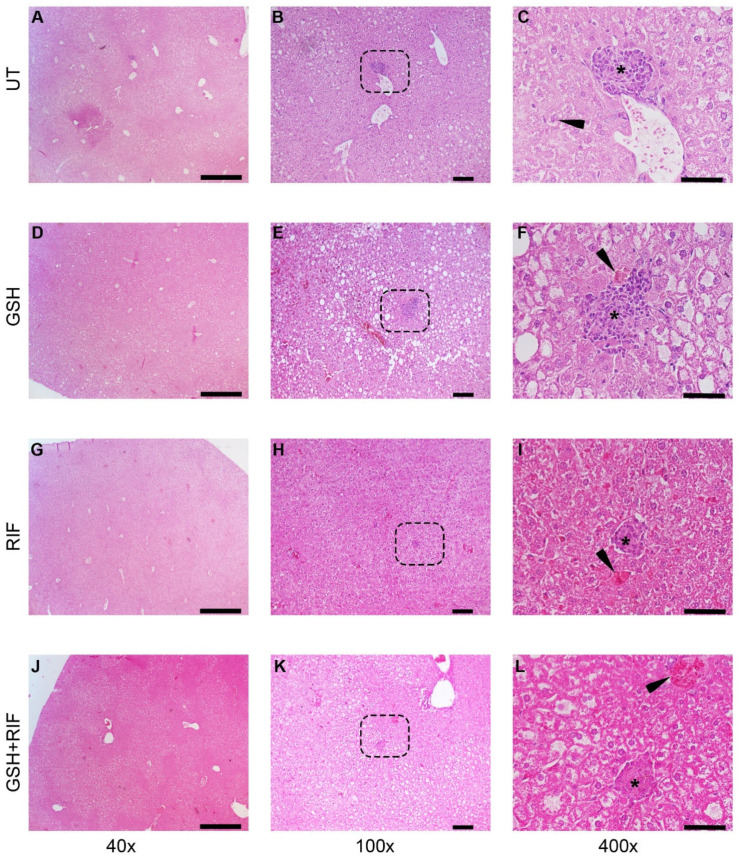
Histopathology of the livers of untreated and treated *M. tb*-infected db/db mice at 8 weeks post-infection/treatment. (**A**–**C**) Untreated (UT); (**D**–**F**) glutathione-treated (GSH); (**G**–**I)** rifampicin-treated (RIF); (**J**–**L**) glutathione plus rifampicin-treated (GSH+RIF). Images (**A**,**D**,**G**,**J**) are at 40× magnification; (**B**,**E**,**H**,**K**) are 100× magnification; (**C**,**F**,**I**,**L**) are 400× magnification. The arrowhead in (**C**) indicates a Kupfer cell with vacuoles in the cytoplasm; arrowheads in (**F**,**I**,**L**) indicate necrotic foci. * Refers to the granulomatous area with different sizes. The dotted rectangles in (**B**,**E**,**H**,**K**) refer to the enlarged sections shown in (**C**,**F**,**I**,**L**), respectively. Scale bar in (**A**,**D**,**G**,**J**) is 500 µm; (**B**,**E**,**H**,**K**) is 100 µm; (**C**,**F**,**I**,**L**) is 50 µm.

At 8 weeks post-infection, distinct granulomas were seen in the livers of all groups of *M. tb*-infected db/db mice, regardless of treatment status (Figure 4).

Liver sections of all groups of *M. tb*-infected db/db mice showed granulomas, comprised mostly of lymphocytes and inflammatory regions surrounding the granulomas. Few necrotic foci of hepatocytes were also observed in these liver sections. 

**Figure 5:**
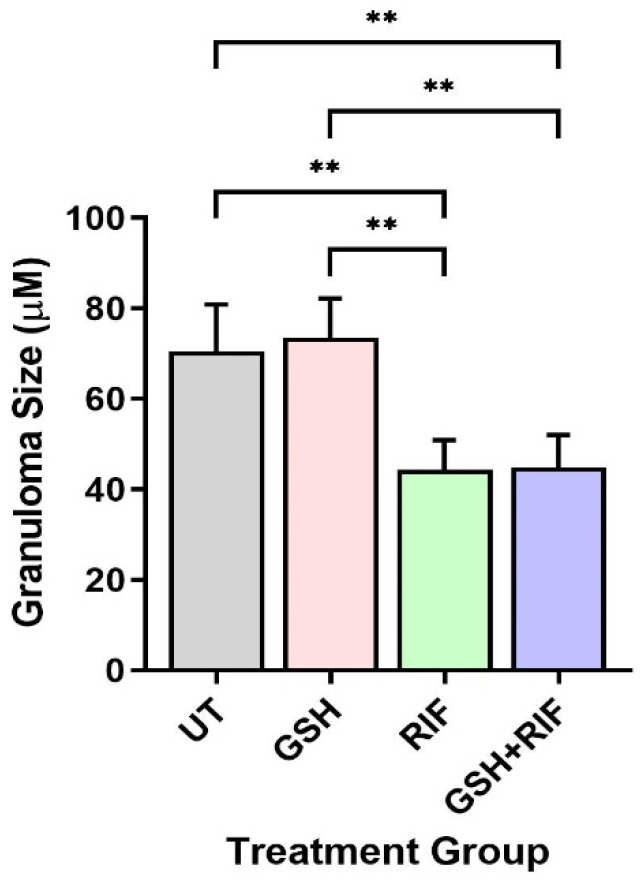
Granuloma size in the livers of untreated and treated db/db mice infected with *M. tb* at 8 weeks post-infection. UT: untreated; GSH: glutathione-treated; RIF: rifampicin-treated; GSH+RIF: glutathione plus rifampicin-treated. Data from 4 animals per group. ** *p* < 0.01 as determined by one-way ANOVA with Tukey’s post-correction.

Next, we measured the granuloma size of db/db mice. As mentioned above, at 4 weeks post-infection, no measurable granulomas were noted in any of the mice livers, regardless of treatment status. In contrast, at 8 weeks post-infection, distinct microscopic granulomas were noted (Figure 5). *M. tb*-infected db/db mice treated with GSH alone did not show any significant difference in granuloma size when compared to the untreated group. However, infected mice treated with RIF either alone or in combination with GSH (i.e., GSH+RIF) had a significantly reduced liver granuloma size compared to the untreated and GSH-only treated mice (Figure 5). There were no significant differences in the liver granuloma size between RIF- and RIF+GSH-treated mice.

Next, we determined the disease score of *M. tb*-infected db/db mice liver with or without various treatments using the Scheuer system. The disease score was graded as follows: Grade 1: minimal inflammation without necrosis, Grade 2: inflammation with mild and focal necrosis, Grade 3: moderate necrosis and severe inflammation, and Grade 4: severe necrosis and severe inflammation [18]. 

**Figure 6:**
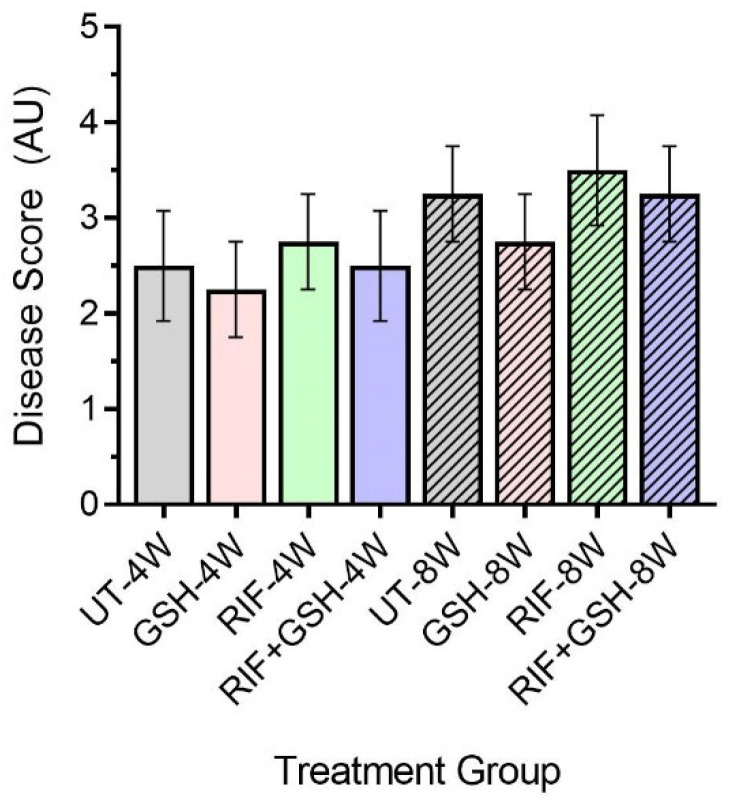
Disease score of livers in untreated and treated db/db *M. tb*-infected mice. UT: untreated; GSH: glutathione-treated; RIF: rifampicin-treated; GSH+RIF: glutathione plus rifampicin-treated—data from 4 animals per group. No statistically significant differences were noted between groups; data were analyzed by one-way ANOVA with Tukey’s post-correction.

Though no distinct granulomas were found in the liver of *M. tb*-infected mice with or without any treatment at 4 weeks post-infection, inflammation was seen in all the infected mice livers, irrespective of treatment. A slightly higher level of inflammation with necrosis was noted in these animals at 8 weeks post-infection. Although the livers of GSH-treated mice had less severe disease scores compared to the untreated group at 4 and 8 weeks, the difference was not statistically significant (Figure 6). None of the treatments showed a significant change in disease score compared to untreated mice at both 4 and 8 weeks (Figure 6).

## 4. Discussion

Approximately 20% of *M. tb* infections worldwide progress to extrapulmonary tuberculosis (EPTB). Extrapulmonary tuberculosis (EPTB) is highly common among immunocompromised individuals, including those with HIV or type 2 diabetes mellitus (T2DM) [19]. This research specifically examines EPTB in the liver. Hepatic TB happens through two different routes: the hematogenous route through the hepatic artery (more common) or the gastrointestinal route through the portal vein [20]. The liver maintains redox homeostasis by generating reduced glutathione (rGSH), hence neutralizing any reactive oxidative species (ROS) generated during an infection [21]. Prolonged infections of *M. tb* have been shown to produce reactive oxidative species, which trigger redox-sensitive transcription factors (Nrf2). Nrf2 binds to the antioxidant response element (ARE) and triggers the expression of ROS-cytoprotective proteins as part of the host’s defense mechanism [22]. ARE controls the enzymes involved in the synthesis and regulation of GSH, including glutamate-cysteine ligase, glutathione-S-transferase (GST), and glutathione synthetase (GS) [23]. The administration of exogenous L-GSH+RIF has been demonstrated to improve the immune response to *M. tb* infection in lung tissue [24]. However, the combined impact of GSH and RIF on liver tissue during an *M. tb* infection remains unclear in vivo [25]. To delve deeper into the existing research, we investigated the influence of L-GSH supplementation in a diabetic (db/db) mouse model infected with *M. tb* alongside RIF treatment. 

A previous research study demonstrated that the supplementation of mice with 40 mM of L-GSH resulted in decreased oxidative stress, increased Th1-promoting cytokines, reduced lung tissue pathology, and a lower overall burden of *M. tuberculosis* during active infection in the lungs of WT C57BL/6 mice [26]. Additionally, other studies have shown that L-GSH supplementation in patients, regardless of their immune status, promoted the granuloma formation of peripheral blood mononuclear cells (PBMCs) derived from patients’ blood in vitro against *M. tuberculosis* infection [27,28,29,30,31]. Our earlier research indicated that PBMCs from T2DM patients infected in vitro with *M. tuberculosis* showed impaired granuloma formation, which allowed for the increased survival and growth of *M. tuberculosis* compared to PBMCs from healthy individuals [30,32]. Importantly, the results from our clinical trial indicate that L-GSH supplementation in T2DM patients restored the GSH levels, decreased the MDA levels, and enhanced the effector immune response to control the growth and survival of *M. tuberculosis* in vitro [28,30,32]. These findings suggest that L-GSH can modulate immune responses that can serve as an adjunctive therapy alongside standard anti-TB drugs for better control of *M. tuberculosis* growth and infection. In this study, we aimed to assess whether treatment with L-GSH, alone and in addition to traditional treatment (rifampicin), would enhance the immune response by measuring pro- and anti-inflammatory cytokines, as well as the total and reduced glutathione in liver tissue. 

In our most recent study, we investigated the *M. tuberculosis* burden in the livers of db/db mice infected via the aerosol route [24]. Compared to untreated *M. tuberculosis*-infected db/db mice, L-GSH treatment did not significantly reduce *M. tuberculosis* survival in the liver at 4 weeks post-infection [24]. However, treatment with RIF led to a large decrease in the number of *M. tuberculosis* in the liver at 4 weeks post-infection [24]. Notably, treatment with a combination of L-GSH and RIF significantly reduced the viability of *M. tuberculosis* in the liver, with the bacterial burden in the L-GSH+RIF group being even lower than that in the RIF-only group [24].

However, the effects of L-GSH supplementation combined with RIF on the cytokine levels and pathogenesis in the liver of *M. tuberculosis*-infected diabetic mice have not been demonstrated. We hypothesized that the combined supplementation of L-GSH and RIF would restore the cytokine balance and reduce inflammation in the livers of *M. tuberculosis*-infected db/db mice. To test our hypothesis, we conducted in vivo *M. tuberculosis* infection studies in db/db mice, examining the hepatic GSH levels, cytokine profiles, and liver pathology in *M. tuberculosis*-infected db/db mice treated with L-GSH, RIF, and the combination of L-GSH+RIF.

Our results indicate that treatment with L-GSH+RIF for 4 weeks of *M. tb*-infected db/db mice caused a substantial increase in the levels of Th1 cytokines (IL-12, IFN-g, and IL-2); pro-inflammatory cytokines (TNF-a, IL-17, and IL-6); and the total and reduced forms of GSH (Figure 1). These results support our previous findings [24] and indicate that exacerbated effector immune responses with L-GSH+RIF treatment for 4 weeks (Figure 1, Figure 3 and Figure 5) lead to improved *M. tb* infection in the livers of db/db mice [24]. 

With the reduction of the *M. tb* burden at 4 weeks in the liver induced by L-GSH+RIF treatment, there is restoration of homeostasis in the immune responses, which is evident from our study findings from L-GSH+RIF-treated db/db mice at 8 weeks post-infection (Figure 2). There was a significant decrease in the levels of Th1 cytokines (IL-12, IFN-g, and IL-2 [Figure 2A–C]) and pro-inflammatory cytokines (TNF-a, IL-17, and IL-6 [Figure 2D–F]) and an increase in the level of the reduced form of GSH (Figure 2I) at 8 weeks post-infection and treatment. This decrease in the levels of Th1 and pro-inflammatory cytokines was more profound in the L-GSH+RIF treatment group when compared to the RIF-only category (Figure 2). 

There were no measurable granulomas at 4 weeks post-infection with or without any treatment (Figure 3). However, at 8 weeks post-infection, distinct microscopic granulomas were noted (Figure 4). Inflammation was seen in all the infected mice livers, irrespective of treatment, at 4 and 8 weeks post-infection. Though no distinct granulomas were found in the livers of *M. tb*-infected mice with or without any treatment at 4 weeks post-infection, inflammation was seen in all the infected mice’s livers, irrespective of treatment. A slightly higher level of inflammation with necrosis was noted in these animals at 8 weeks post-infection. Although not statistically notable, the livers of L-GSH-treated and L-GSH+RIF-treated mice had less severe disease scores compared to the untreated and RIF-treated mice, respectively, at 4 and 8 weeks post-infection and treatment (Figure 6). Our study findings indicate that L-GSH supplementation may confer immunotherapeutic effects during EPTB by mitigating inflammation. 

At both termination points (4 and 8 weeks), there was a significant increase in the total GSH with L-GSH treatment alone. However, L-GSH+RIF did not result in a significant increase in the total GSH at 4 and 8 weeks. For reduced GSH, L-GSH resulted in a significant increase at both 4 and 8 weeks, whereas L-GSH+RIF resulted in a significant increase at 8 weeks only. The mechanisms of these combination- and time-dependent levels of glutathione remain unclear. 

Despite the RIF and L-GSH+RIF treatments proving a significant change in the cytokine levels and granuloma sizes, the disease scores for both treatments were not significantly different from the untreated sample. Traditionally, it is believed that there is a direct and proportional correlation between bacterial load and the granulomatous response, which serves as a marker of disease pathology and determinant of the disease score. However, this notion has been challenged by several seminal studies on the non-human primate model of TB [33,34,35]. These studies demonstrated the discordance between bacterial load and markers of disease pathology, indicating that they are independent entities [33,34,35]. Consequently, some granulomatous lesions were observed without any bacterial presence, while others showed variable bacterial loads within the same animal and across different animals [33,34,35]. Consistent with these findings, our study did not identify any correlation between bacterial load and disease pathology in the livers of mice infected with TB. Our study further enhanced that liver pathology and bacterial load in mice during TB infection can be independently regulated. 

Our study findings in diabetic mice indicate that GSH has both protective and additive effects against *M. tb* infection. Therefore, our long-term goal will be to conduct a clinical trial in a TB endemic country to test the efficacy of GSH administered along with anti-TB drugs in diabetic patients and active pulmonary TB in preventing both dissemination of infection to extrapulmonary sites and causing the complete resolution of *M. tb* infection in the liver.

## Data Availability

The data supporting reported results can be obtained from the corresponding author (V.V.) upon formal requisition.
